# MPPT mechanism based on novel hybrid particle swarm optimization and salp swarm optimization algorithm for battery charging through simulink

**DOI:** 10.1038/s41598-022-06609-6

**Published:** 2022-02-17

**Authors:** Idriss Dagal, Burak Akın, Erdem Akboy

**Affiliations:** 1 Electrical Engineering, Yildiz Technical University, 34220 Namık Kemal Esenler, Istanbul, Turkey; 2 Electrical Engineering, Yildiz Technical University, C-318 Davutpaşa Campus, Istanbul, Turkey; 3 Electrical Engineering, Yildiz Technical University, C-18 Davutpaşa Campus, Istanbul, Turkey

**Keywords:** Electrical and electronic engineering, Batteries

## Abstract

In this paper, a battery charging model is developed for solar PV system applications. As a means of photovoltaic power controlling system, buck-boost converter with a Maximum Power Point Tracking (MPPT) mechanism is developed in this paper for maximum efficiency. This paper proposed a novel combined technique of hybrid Particle Swarm Optimisation (PSO) and Salp Swarm Optimization (SSO) models to perform Maximum Power Point Tracking mechanisms and obtain a higher efficiency for battery charging. In order to retrieve the maximum power from the PV array, the Maximum Power Point Tracking mechanism is observed which reaches the maximum efficiency and the maximum power is fed through the buck-boost converter into the load. The buck-boost converter steps up the voltage to essential magnitude. The energy drawn from the PV array is used for the battery charging by means of an isolated buck converter since the buck-boost converter is not directly connected to the battery. The Fractional Order Proportional Integral Derivative (FOPID) controller handles the isolated buck converter and battery to enhance the efficiency obtained through the Maximum Power Point Tracking mechanism. The simulation results show higher steady efficiency by using the hybrid PSOSSO algorithm in all stages. The battery is charged without losing the efficiency obtained from the hybrid PSOSSO algorithm-based Maximum Power Point Tracking mechanism. The higher efficiency was obtained as 99.99% at Standard Test Conditions (STC) and 99.52% at PV partial shading conditions (PSCs) by using the new hybrid algorithm.

## Introduction

Solar energy and photovoltaic (PV) systems especially are widespread due to their easy installation process. The PV cells are used for solar radiation conversion into electrical energy. The PV systems efficiency has been improved with the Maximum Power Point Tracking (MPPT) mechanism and non-linear electrical characteristics solutions. With power electronic devices, solar panel efficiency is thus increased along with the output power. The conversion efficiency that depends on the matrix operating voltage has been enhanced by the MPPT algorithm which is widely used in PV applications. Therefore, the MPPT algorithm helps to obtain the greatest efficiency with the least cost. To design the MPPT mechanism, the DC–DC converter is exploited^1^. In this study, the buck-boost converter is selected among other various DC–DC converter types. The buck-boost converters are referred to as inverting and non-inverting types and they can give a maximum output voltage range with respect to the input voltage. Based on the switching semiconductor and duty cycle, the output voltage is adjustable. There are no consequences seen if the power supply is isolated from the load circuit. Since the diode polarity and the power supply are simply reversed, and the switch is either on the supply side or the ground side. A step-down or buck converter associated with the step-up or boost converter constitutes a buck-boost converter with the same input polarity and the output can be higher or lower. The MPPT algorithm usage and suitable optimization algorithms are suggested to attain maximum efficiency^2^. To reach the PV panel maximum power point, the hybrid algorithm is suggested in this study. Particle swarm optimization is used in various studies to attain efficiency^3^. However, in this study, the PSO is combined with the Salp Swarm optimization algorithm (SSA) to enhance the application to be more efficient. Furthermore, the battery supply is connected for charging which is not directly connected to the buck-boost converter. Hence the isolated buck converter is used in this study^4^ and there are no consequences that occur if the power supply is isolated from the load circuit. For the switched-mode power supply, the isolated buck converter is used. Thus, the issues of previous studies’ approaches have at least diminished. The analog input was converted to analog output by an isolated buck converter which comprised the capabilities of digital data transferring among the main circuit and isolated buck converter. In the PV array system, the Fractional order PID controller has been used to regulate the output voltage of the DC-DC converter where PID is the Proportional integral derivative. For controlling the linear and non-linear systems in a wide range area, which is compared with classical PID controller, FOPID controller is preferable. FOPID controller gives more flexibility, robustness, and quality in controller design^5^. Similarly, this study^6^ explored an efficient strategy that uses the MPPT algorithm to raise the efficiency of the PV panel. This study recommends Fuzzy Logic Controller (FLC), as well as the Modified, Shuffled Frog Leaping Algorithm (MSFLA) for optimal parameter tuning of the controller thereby following MPP. About this, an effective theoretical outline has been introduced for the proposed algorithm to validate its efficacy under environmental change conditions. The results revealed that an efficient power flow amongst the grid and the combined Energy Systems has been accomplished. That is related to augmented stability and the capability of transient mode. Besides, this article^7^ explored power quality control in the smart AC/DC microgrids (hybrid) on the real-world issues of the microgrids in terms of power quality. A Cuckoo Search Optimization (CSO) based on the MPPT algorithm has been employed to afford the best fitness function (FF) to vary the duty cycles. The simulation results have also been presented for supporting the proposed concept. Besides, this study has not been considered for the partial shading conditions. This is yet to be developed. Besides, this study^8^ proposed a Particle Swarm Optimized-Proportional Integral (PSO-PI) controlled DC-DC for testing the battery charging controller and the MPPT algorithm. The results revealed that the efficiency of the proposed method is improved. Additionally, this paper recommended an Artificial Bee Colony (ABC) algorithm for Maximum Power Point Tracking (MPPT) of the PV system by using a DC-DC converter. The proposed methodology has been compared with the existing systems. The outcomes revealed the efficacy of the proposed methodology^9^. Hence, this study aimed at increasing the efficiency of battery charging from PV by the MPPT algorithm based on the proposed novel hybrid PSO (Particle Swarm Optimisation) and SSO (Salp Swarm Optimisation). This study is implemented in Simulink to assess its performance. In addition, various comparative analysis is performed to prove the efficiency of the proposed methodology. The main contribution of this study involves:

- To perform the MPPT process in the PV (Photovoltaic) system combined with the Buck-Boost Converter (BBC) using the proposed novel hybrid PSO (Particle Swarm Optimisation) and SSO (Salp Swarm Optimisation) to increase the efficiency of battery charging.
- To perform battery charging through the Fractional Order Proportional Integral Derivative Controller (FOPID) and Isolated Buck Converter in Simulink without affecting the efficiency of MPPT.
- To validate the efficiency of the proposed methodology by comparing it with the existing methods in terms of periodic tuning, tracking accuracy, steady-state oscillation, tracking speed, algorithm complexity, energy, peak power, average power, and efficiency. Moreover, this study aims to take the partial shading conditions into account through comparative analysis.

### Paper organisation

For the paper organization, the proposed study consists of the introduction in section “Introduction”, many related works in section “Related works”, the proposed work methodology based on PV array, MPPT technique, buck-boost converter, isolated buck converter, hybrid PSO, and SSO algorithm has been elaborated in section “Methodology”, the results and discussion are depicted in section “Results and discussion” and finally, the paper is concluded in section “Conclusion”.

## Related works

The following section describes the review of literature related to the buck-boost converter, optimization algorithm, MPPT mechanism, isolated buck converter, and FOPID controller. For maximum power searching, the Perturb and observe (PO) MPPT process has been imposed for the battery charging. The system with MPPT shows greater output power compared with the system without MPPT. The efficiency obtained was 90.56 percentage and maximum power has been transferred to the battery^10^. Furthermore, the MPPT technique used to extract maximum power in a photovoltaic system is also illustrated in this^11^ study. For the photovoltaic system incorporated through a Z-source inverter, the improvised Particle Swarm Optimization (PSO) depends on the MPPT technique that has been involved in this paper. When the maximum power point has been placed, the steady-state oscillation has diminished. This proposed system has tracked the MPP efficiently even though extreme environmental conditions occur like large fluctuations and partial shading conditions related to the abrupt change in the temperature and the irradiance. Correspondingly, for the unshaded PV systems, the maximum power is extracted and exhibited only one power peak in the P–V curve. Various peaks are generated from the partial shading conditions (PSCs) like multiple local maximum power points (LMPP) and global maximum power points (GMPP). In the searching process, experimental methods like PSO or the Gray Wolf Optimization (GWO) identified the GMPP. The GMPP changes its position and value if the PSC value is changed. However, the GWO or PSO has not caught this GMPP value during the previous GMPP search area and only the searching agents are searched. Based on the PSC changes the searching agents should be re-initialized. As per the discussion point of view, this^12^ study adopted the GWO- Fuzzy logic controller integrated technique that shows better performance in the tracking of dynamic GMPP. Moreover, the output power oscillations have been minimized by this integration^13^. This study^14^ focused on the photovoltaic systems, in which a three-port switching boost converter considered as a non-isolated converter has been applied and the network of switching boost characteristics is combined. These ports have exhibited the boost, buck, and buck-boost converters characteristics by controlling three degrees of freedom. The three-port converter has been developed with the basic feature, low cost, and small size. In various working modes, the system power flow has been verified^15^. The photovoltaic system feasibility has been shown after evaluation. Additionally, a sliding mode MPPT controller has been designed in this^16^ research for the PV systems in the atmospheric rapid change conditions. The gains of optimal sliding mode controller (SMC) have been identified by the PSO algorithm^17^. In the online mode operation, the optimal sliding mode controller (SMC) gains are used for MPPT step drive whereas, in the offline mode, the requirements for various sets of optimal SMC gains testing can result in some optimum values. A maximum power point with better tracking speed, low overshoot, low ripple, and low oscillation has been effectively tracked in both fast and slow-changing atmospheric conditions. Furthermore, a DC–DC regulated buck-boost converter has been developed for PV systems in another^18^ research. For easy implementation, the MPPT based incremental conductance (INC) has been selected. The suitable output voltage has been introduced by the buck-boost converter that ensures better transfer of the energy. The MPPT algorithm-based PSO is used to overcome these limitations and irradiation imperfection has been identified in^19^ research. The adaptive PID is used to control the buck converter input voltage.

From the design point of view, the model reference adaptive control (MRAC) is similar to the traditional PID structure. To reach PV array maximum power, the PSO with high tracking speed and high tracking accuracy is helpful according to this study. Particularly for the solar PV system, the DC–DC converters act as a major role in the applications of renewable energy. To supply suitable DC voltage with particular loads, various topologies of incorporated DC–DC converters with solar PV systems have been used. The drawbacks like low voltage, unstable and unregulated voltage generation have been solved using these types of DC–DC converters (buck, boost, and buck-boost converters). For maximum output power, the MPPT algorithm is used as discussed above. In this study, it is suggested that the DC–DC buck-boost converter incorporated with a solar PV system shows higher efficiency compared to other converters. The solar PV power generation growth supported by limited cost with enhanced efficiency is obtained by these topologies^20^. At various DC voltage gains and different power levels of the converter, the higher power conversion efficiency has been achieved. The asymmetrical pulse width modulation and the phase-shift modulation have been connected. Through resonant network and in case of discontinuous current both control methods are assessed. In steady-state analysis and buck mode, the quasi-z source Series resonant converter operating principle has been explained in this study. From PV system, in different operating conditions, the buck converter input voltage is essential for a higher energy range. The new isolated buck-boost converter is used for the load regulation and ultra-wide input voltage without any decreases seen in the efficiency^21^. The load frequency control in power system operation is considered a major issue. Compared with the PID controller, the FOPID controller shows more flexibility. This study utilized the FOPID controller merits and gases Brownian motion optimization (GBMO) technique for load frequency to solve the load frequency control issue^22^.

## Methodology

**Figure 1:**
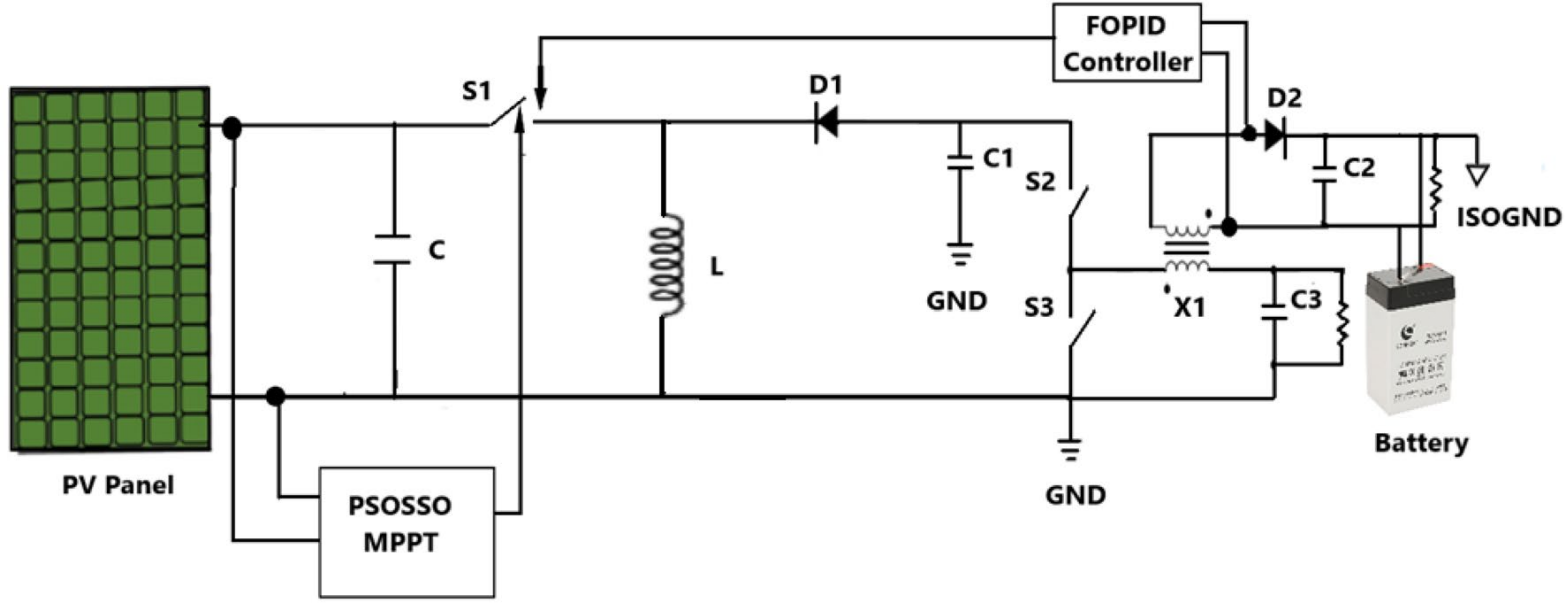
The circuit diagram of the proposed PSOSSO algorithm.

In this study, the implemented solar panel with the buck-boost converter is used to obtain the maximum power for charging the battery. With the help of an isolated buck converter and the fractional-order proportional integral derivative (FOPID) controller, the required current is transferred to the battery for charging the battery since the buck-boost converter is not directly connected to the battery for charging and the hybrid PSOSSO algorithm is used to optimize the PV output power. The circuit diagram of the proposed work is shown in Fig. 1.

The power variation exists and to get the maximum power, the MPPT mechanism is used. To execute the MPPT mechanism the novel hybrid technique namely Particle Swarm Optimization (PSO) combined with Salp Swarm Optimization (SSO) is developed in this study. Similarly, to handle the isolated buck converter and the battery system, the FOPID controller is used.

### PV module equivalent circuit model

**Figure 2:**
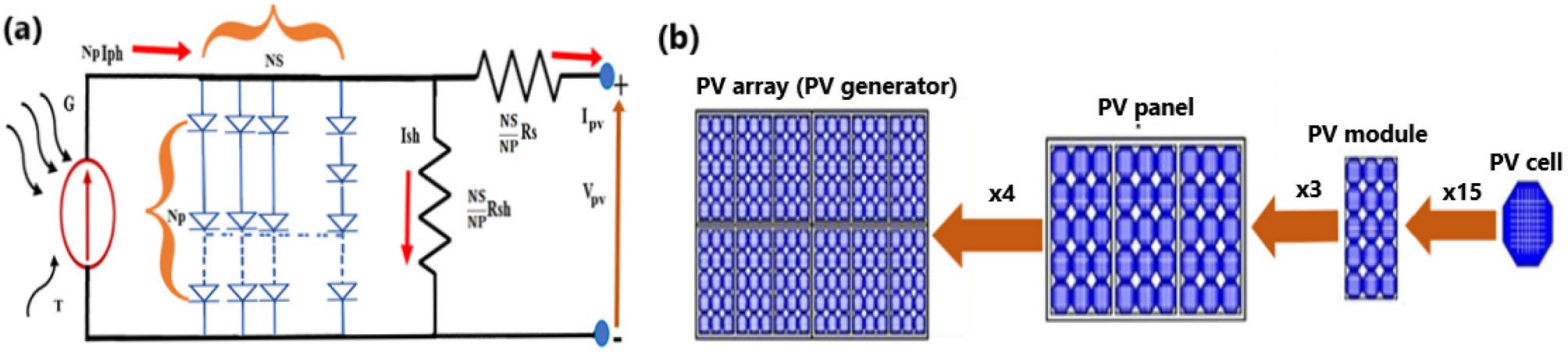
PV module equivalent circuit (**a**). photovoltaic array pyramid (**b**).

The PV module equivalent circuit Fig. 2a is characterized by parallel cells (Np) and similar series cells (Ns). The parallel connection is seldomly used^23^. The series connection increases the output voltage of the PV module. Based on the number of cells, photon current, diode reverse saturation current and ideal factor increase the Ns times^23^. Photovoltaic cell means a tiny device of PV solar configuration system^24^ or a unit made of semiconductor material (doped silicon ingots) based on PV solar construction. Photovoltaic module or panel is set, or group linked of cells. An array is a series or/and parallel connection of modules or a group of modules. In the PV system, the amount of power to be generated is related to the size of the PV panel (meaning the number of cells, modules, or array). PV cell, PV module, and PV array can produce a respectively small amount of power (few watts), medium power (hundreds of watts), and high amount of power range from (kilowatts to megawatts). The price of the PV system is commensurate with the amount of the required power. Figure 2b below shows the distinction of cells, module, and array. The high output voltage is acquired by connecting the solar cells in series whereas a high current is realized by connecting the solar cells in parallel or increasing their dimensions area^24^. Thin-film PV panels have greatly increased worldwide market in the previous decades because of their small price of PV production^25^. In low irradiance conditions, the amorphous silicon thin film with lower grade and price demonstrated better performance compared to mono and polycrystalline solar panel types.

1 $$I_{PV} = N_{P} I_{Ph} - N_{P} I_{d} \left( {exp\left[ {\frac{{q\left( {\frac{{V_{PV} }}{{N_{S} }} + R_{S} \frac{{I_{PV} }}{{N_{P} }}} \right)}}{{kT_{C} n}}} \right] - 1} \right) - \frac{{\left( {\frac{{N_{S} V_{PV} }}{{N_{S} }} + IR_{S} } \right)}}{{R_{Sh} }}$$

2 $$R_{S} \left( {Total} \right) = \frac{{N_{S} R_{S} }}{{N_{S} }}$$

3 $$R_{Sh} \left( {Total} \right) = \frac{{N_{P} R_{S} }}{{N_{S} }}$$

**Figure 3:**
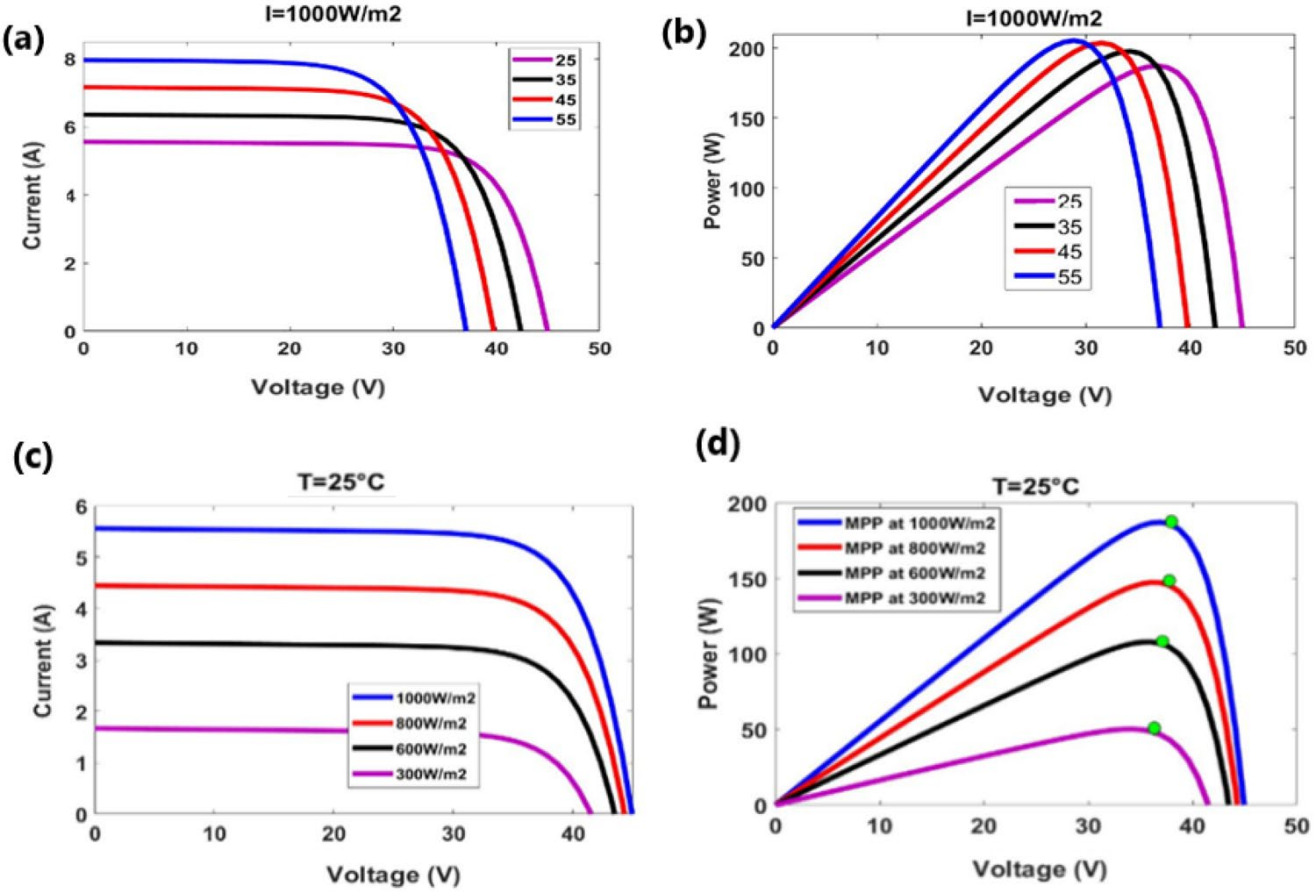
Depicted the I-V, P-V curves with varying irradiance and temperature.

The graph presents the I–V curve indicating a slight increase of short-circuit current and a decrease of open-circuit voltage corresponding to an increase of temperature values Fig. 3a. The graph presents the P–V curve indicating the decrease of open-circuit voltage corresponding to an increase of temperature values Fig. 3b. The graph presents the I–V curve indicating the decrease of short circuit current corresponding to the decrease of irradiance values Fig. 3c. The graph presents the P–V curve indicating the decrease of maximum power point corresponding to the decrease of irradiance values Fig. 3d.

#### Buck converter

**Figure 4:**
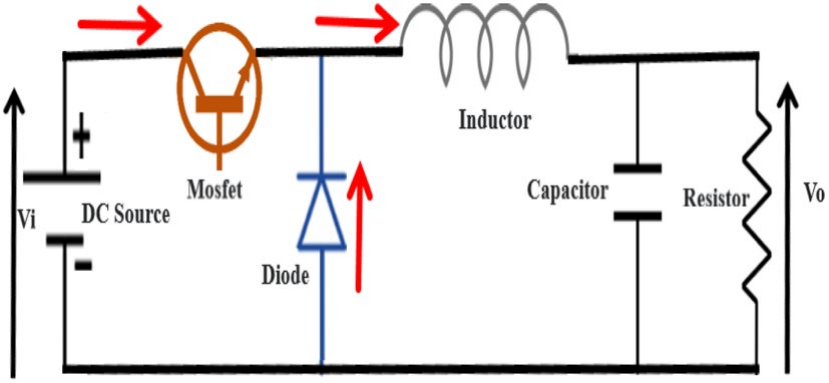
Schematic diagram of Buck Converter.

The Buck converter basic circuit (Fig. 4) components are composed of a Mosfet switch known as a main or controlled switch and a diode switch known as a secondary or uncontrolled switch are used to direct the energy flow unidirectionally from the input to output. The inductor and the capacitor are used as energy-storing or transferring devices. They are also used as current and voltage smoothing devices as well.

The input voltage and output voltage relationship

4 $$V_{in} = DV_{0} \;\;\;\;\;\;\;\;{\text{with}}\;D\;{\text{the}}\;{\text{ duty }}\;{\text{cycle}}$$

The peak-to-peak inductor ripple current is deduced as following

5 $$\Delta L = D\left( {1 - D} \right)\frac{{V_{0} }}{{f_{p} L}}$$

The inductor average current value which is equal to the dc load current

6 $$I_{{Lav}} = \frac{{V_{0} }}{R} = I_{0}$$

The maximum peak to peak inductor ripple current

7 $$\Delta L_{\max } = \frac{{V_{0} }}{{4f_{p} L}}$$

#### Working principle

The working principle of the proposed work is:

- In the tracking algorithm, for fast response, the number of the iteration is reduced or kept lower.
- We control the duty cycle with 4 searching agents, all the agents should optimize and successfully accelerate and give each selected agent the chance of participating or migrating, thus the salp swarm optimization (SSO) algorithm is chosen for this purpose.
- To get an accurate value of the selected 4 searching agents, the particle swarm optimization (PSO) algorithm is chosen for this purpose.
- By combining the SSO and PSO algorithm and with the help of Eqs. (8)–(13), the salp swarm optimal method will find the search zone (range limit), that zone is a place where the target optimal point is located i.e., the required duty cycle is somewhere in that zone.
- By applying the PSO optimal method, the direction is found to attain the required duty cycle.
- By selecting the specific zone area, and applying the PSO for direction search, we can attain the nearest most value of the required duty cycle in the first cycle of the simulation.

#### Problem formulation

The PSO has a premature local minima stagnation (stucking) problem and the SSO has also diversification (exploration of search space) and intensification (exploitation of the solution found)^26^ issues with multiple functions global optimum search. Under partial shading conditions (PCSs), multiple local maximum power points (LMPP) occur in the power–voltage (P–V) characteristic of the PV solar system which is difficult to overcome by the conventional PSO and SSO optimization algorithms. However, a proper balance of exploration and exploitation is required for the SSO algorithm to accurately explore the search space and move gradually toward the global optimum. The efficacy of the SSO algorithm is improved by updating the position of the leader particle or swarm with respect to the food source only, inferring with the solution obtained so far and the leader particle always exploits and explores the space around it. The stagnation issue in the local point is prevented by the gradual movement of the followers of the SSO algorithm. Therefore, the hybrid PSOSSO algorithm is proposed to solve the above-mentioned issues. The primary objective of the problem is to track the global maximum operating point (GMOP)^27^ under various irradiance changing conditions by operating the DC-DC converter at an optimal duty ratio.

Here is the formulation of the problem for the maximum power point tracking (MPPT) mechanism:

1. Optimizing the output power of PV panel which is a function of the duty cycle $D$ presented as:
  8 $$P_{PV} \left( {D_{i}^{k} } \right) \ge P_{PV} \left( {D_{i}^{k - 1} } \right)\;\;\;\;\;\;\;{\text{and }}\;{\text{considered }}\;{\text{as }}\;{\text{the }}\;{\text{objective }}\;{\text{function}}$$

Subject to:

9 $$D_{\min } \le D \le D_{\max } \;\;\;\;\;\;\;{\text{considered}}\;{\text{ as}}\;{\text{ its }}\;{\text{constraints}}$$

$D_{\min }$ and $D_{\max }$ are the minimum and maximum values of the converter duty ratio respectively. In practical implementation, these values are taken as 10% to 90% respectively.

In this study, the DC–DC converter’s duty cycle *D* for 4 searching agents and the particle position $x_{i}^{k}$ has been presented during the implementation of the PSO in the power tracking MPPT mechanism. The value of the duty cycle is updated by the variation of 4 cycle value with the velocity of the particle $\vartheta_{i}^{k}$(meaning the converter and the optimal method are mathematically connected by the converter duty cycle $D$ and the particle’s speed $\vartheta_{i}^{k}$ of the optimization method).

#### Particle Swarm optimization

The particle swarm optimization (PSO) is a stochastic method with enthused computational research and optimization technique that symbolizes the portrayal movements of organisms based on an intelligent global behavior of birds’ flock or fish school 2015^27^. In^28^, the PSO optimizes many continuous and discreet problems. The PSO is used to solve some optimization problems, parameters called particles are used in the algorithm implementation, the particles are objects or entities randomly dispersed in the search space and searched for the best position to be optimized. Each particle controls its motion by combining the history of its current and previous best fitness locations with other groups or members of the swarm in random perturbation of the search space^29^. The cos function for the calculation of each particle within the geometric of the space. The population is denoted as a factor that may influence the performance of the PSO algorithm, many populations promote the diversity of the swarm and its exploration capability and increase the premature convergence chance and the computational labors^30^. The PSO algorithm encompasses local and global forms, the optimal position pbest of its own and gbest of the swarm optimal position that the track of the particle is called global form whereas; when the particles fail to track the swarm gbest position and its own pbest optimal position, but they track in its neighborhood topology other particles nbest optimal position is called the local form^31^. Although the PSO is an outstanding technique to solve the optimization problems and is considered as one of the newest and the most effective technique in the epoch of probabilistic metaheuristics, it has its drawback of stucking in local minima, to solve this premature convergence problem^32^, proposed new master–slave swarms shuffling evolution algorithm based self-adaptive particle swarm optimization (MSSE-SPSO) and^33^ introduced that its performance is affected by the size of the population, inertia weight, acceleration constants and the design of updating velocity strategies. Therefore, researchers work to improve it by altering the initialization, introducing new parameters, changing the local and global best particles based on their mutation operators, or describing the different techniques of inertia weight to change its performance^34^. The proposed multi-swarm particle optimization (MPSO)^35^, has good performance in quality and robustness of the solution than the self-adaptive particle swarm optimization (SPSO).

##### PSO algorithm working principle

**Figure 5:**
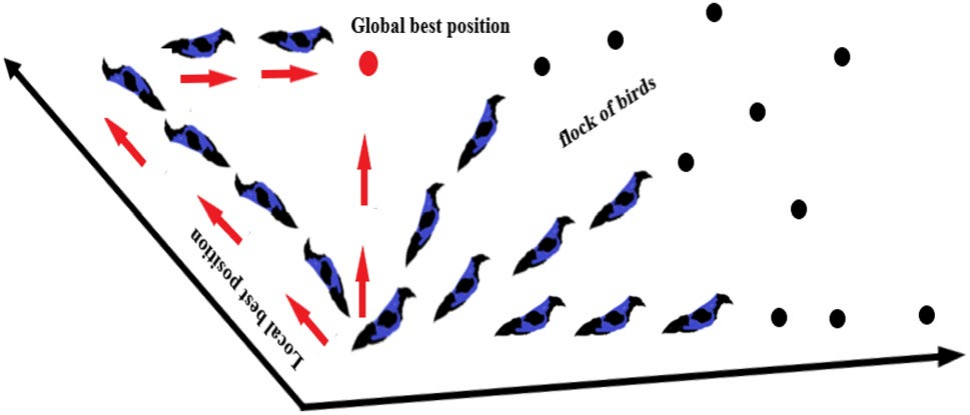
The flock of birds in their random movement for the best optimal position search.

The PSO technique works on the principle of natural creatures’ civilizations behavior. The common example of bird flocking, birds flying through the search space of fetched food in random movement, their direction is followed up by a member of a group that position deemed the nearest distance to their targeted food place. The birds called swarms or particles freely move without leadership basis, they reach their global best position Fig. 5 through message by referring to members with the improved situation and that recall their previous condition or local best position. A bird with better condition, announces its current place to the other flocks or groups and they will orient simultaneously themselves toward the global optimum solution and then the process resumes until it has reached the targeted food or prey source. Every motion is characterized by its position and velocity mentioned in Eqs. (10) and (11).

The status of each particle is characterized by its position and velocity where Eqs. (10) and (11) indicated respectively the updating velocity and the position.

10 $$\vartheta_{i}^{k + 1} = \omega \vartheta_{i}^{k} + c_{1} r_{1} \left( {pbest_{i}^{k} - X_{i}^{k} } \right) + c_{2} r_{2} \left( {gbest^{k} - X_{i}^{k} } \right)$$

11 $$x_{i}^{k + 1} = x_{i}^{k} + \vartheta_{i}^{k + 1}$$

where:

$pbest_{i}^{k} - X_{i}^{k}$ is designated as cognitive or personal components; $gbest^{k} - X_{i}^{k}$ is designated as global or social components;$c_{1}$ and $c_{2}$ are accelerating constant; *ω* is the inertia weight factor; $r_{1}$ and $r_{2}$ are two numbers range.

[0 1] generated erratically ; $pbest_{i}^{k}$ is the best $i$ particles position achieved for iteration index and can be expressed as $gbest^{k} = [X_{1}^{gbest} ,X_{2}^{gbest} ,X_{3}^{gbest} , \ldots \ldots \ldots .,X_{N}^{gbest} ]$;$gbest^{k}$ is the best particle position and can be expressed based on swarm’s experience as:$gbest^{k} = [X_{1}^{gbest} ,X_{2}^{gbest} ,X_{3}^{gbest} , \ldots \ldots \ldots .,X_{N}^{gbest} ]$.

$c_{1}$ and ${\mathrm{c}}_{2}$ generate a balance among the individual components and the global components in the velocity expression (10).

When the particles are held to turn with their initial velocity until reaching the searching space boundary: $c_{1}$ and $c_{2}$ equal to zero and Eq. (10) becomes $\vartheta_{i}^{k + 1} = \omega \vartheta_{i}^{k}$.

When particles separately hovered around their searching space, they cannot obtain the optimal global solution due to no interaction with other neighbors. In this situation, $c_{1}$ $\mathrm{is greater than zero}$ and $c_{2}$ $\mathrm{is equal to zero}$, the Eq. (10) becomes $\vartheta_{i}^{k + 1} = \omega \vartheta_{i}^{k} + c_{1} r_{1} \left( {pbest_{i}^{k} - X_{i}^{k} } \right)$.

When the particles are captivated to a unique point ($gbest$) which is not verified at each stage of time, in this case: $c_{1}$ is equal to zero and $c_{2}$ is greater to zero, the Eq. (10) becomes $\vartheta_{i}^{k + 1} = \omega \vartheta_{i}^{k} + c_{2} r_{2} \left( {gbest^{k} - X_{i}^{k} } \right)$.

When all the particles converge towards the average values of Pbest and gbest $c_{1}$ and $c_{2}$ are equals and the Eq. (10) becomes $\vartheta_{i}^{k + 1} = \omega \vartheta_{i}^{k} + c_{1} \left[ {r_{1} \left( {pbest_{i}^{k} - X_{i}^{k} } \right) + c_{2} \left( {gbest^{k} - X_{i}^{k} } \right)} \right]$.

When the particles are greatly migrating towards to pbest, therefore; $c_{1}$ is greater than $c_{2}$ and in contrast, when the particles are greatly influencing towards to gbest, $c_{2}$ is greater than $c_{1}$ causing premature optima trapped. In the next cycle, we can attain hence, the maximum output. The PSO technique syndicates both the particle self-experience and its informants’ experience to balance the exploitation and the exploration^36^.

#### Salp Swarm optimization

**Figure 6:**
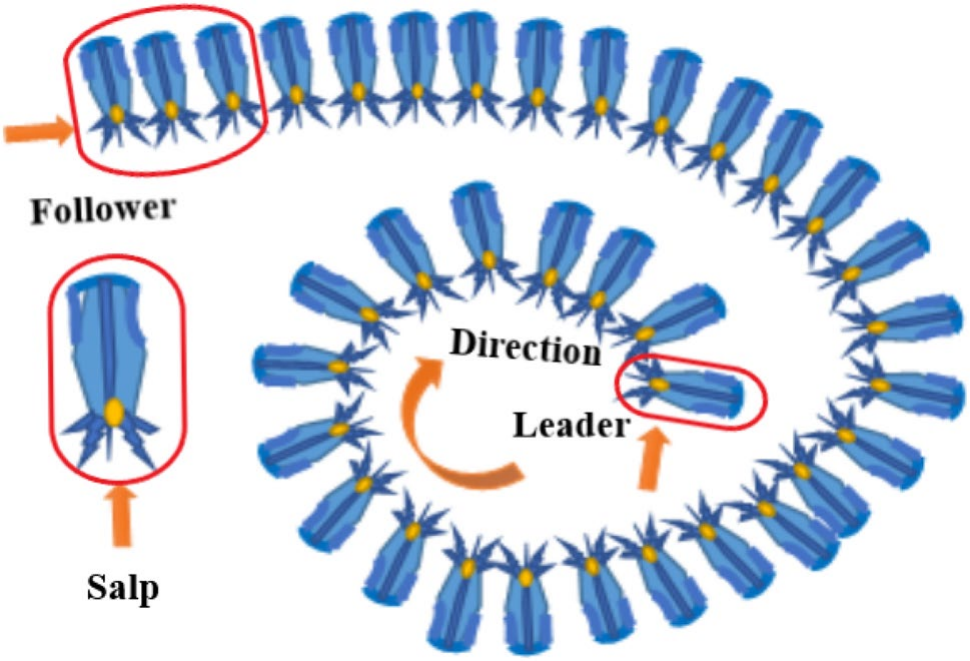
Structure of salp chain.

For solving the optimization problems in salp swarms no mathematical model is presented. In case for solving the optimization problems the swarms of ants, fishes and bees have modeled. The leaders and followers are categorized and formed into groups Eq. (12). The swarm is guided by the leader in which the followers follow each other Fig. 6. In n-dimensional search space, the salp position is defined and stored in the two-dimensional matrix x. In the search space, there is a food source F and to update the leader position the following Eq. (12) is proposed,

12 $$X_{j}^{1} = \left\{ \begin{gathered} F_{j} + c_{1} \left( {\left( {\mu b_{j} - lb_{j} } \right)c_{2} + lb_{j} } \right)c_{3} \ge 0 \hfill \\ F_{j} - c_{1} \left( {\left( {\mu b_{j} - lb_{j} } \right)c_{2} + lb_{j} } \right)c_{3} < 0 \hfill \\ \end{gathered} \right.$$

From Eq. (12), ${x}_{j}^{1}$ is the first salp leader position in the jth dimension, food source position is ${F}_{j}$ in jth dimension, upper bound and lowers bound is $u{b}_{j}$ and $l{b}_{j}$ respectively in jth dimension, the random numbers are ${c}_{1}$, ${c}_{2}$ and ${c}_{3}$. With respect to the food source, only the leader updates the position. In SSA, ${c}_{1}$ is considered a significant parameter since the exploitation and exploration is balanced by it expressed as,

13 $$c_{1} = 2e^{{^{{ - \left( \frac{4l}{L} \right)^{2} }} }}$$

From Eq. (13), the current iteration is $l$ and the maximum no. of iterations is $L$. ${c}_{2}$ and ${c}_{3}$ parameters are uniformly generated random numbers in [0,1] interval. If the next position in the jth dimension is towards the size of step, positive or negative infinity. To update the followers’ position Newton’s law of motion is used,

14 $$x_{j}^{1} = \frac{1}{2}at^{2} + v_{o} t$$

From Eq. (14) with $i \ge 2$,$x_{j}^{1}$ is the ith salp follower position in jth dimension, time $t$, initial speed $v_{o}$. Whereas $v = \frac{{x - x_{o} }}{t}_{{}}$ $a = \frac{{v_{final} }}{{v_{{o_{{}} }} }}$ and. Since the optimization, time is iteration, the divergence among the iterations = 1, and for $v_{o} = 0$ the Eq. is,

15 $$x_{j}^{i} = \frac{1}{2}\left( {x_{j}^{i} + x_{j}^{i - 1} } \right)$$

From Eq. (15), $i \ge 2$ and $x_{j}^{i}$ is the ith follower salp position in jth dimension.

### Hybrid PSO and SSO

Novelty is the hybridization of Salp Swarm Optimization (SSO) and Particle Swarm Optimization (PSO), the SSO will initially work, and it will find the particle position, Pbest, and Gbest. Further, PSO will run with these values (Position, Pbest, and Gbest from SSO), then the PSO returns to the final particle position, Pbest, and Gbest values. The SSO will search the larger area and PSO will search inside the area found by SSO.

The following algorithm describes the hybrid PSO and SSO optimization techniques in Table 1 below.

**Table 1:** Proposed PSOSSO algorithm flowchart.

Algorithm
**Salp Swarm Optimization Algorithm (SSO)**
1. Initialize the duty cycle (dc) near the ub
2. Calculate the fitness of each search agent (salp)
3. Find the best value and best position
4. F = the best search agent (best position)
5. i = searching agent
6. Update C1 by Eq. (13) for each salp (xi)
**Particle Swarm Optimization Algorithm (PSO)**
7. Initialize the PSO parameters,
8. Inertia Weight W,
9. Personal Learning Coefficient c1
10. Global Learning Coefficient c2
** Hybrid** **Algorithm (PSOSSO)**
11. Update the PSO velocity by Eq. (10)
12. Update PSO position by Eq. (11)
13. If (i == 1)
14. Update leading salp position Eq. (12)
15. Else
16. Update salp follower position Eq. (15)
17. End
18. Amend the salps based on the upper and lower bounds of variables
19. Velocity = salp position divide by the PSO velocity
20. End
21. Update the position dc(i) = dc(i) + velocity

At first, the duty cycle near the ub is initialized. Then, the fitness of each search agent is calculated. Subsequently, the best value and best position are determined. Followed by this c1 is updated as per step 6. After this, the PSO parameters are initialized. After a series of steps, the velocity is updated as per step 11. The leading salp and salp follower positions are updated. Later, the salps are amended based on the upper and lower bound of variables. Finally, the position dc (i) is updated as per step 21.

## Results and discussion

In this section, five (5) cases with various situations were studied.

Overall, extensive analyses have been achieved on MATLAB/Simulink application under various patterns of partial shading conditions (PSCs).

The proposed PSOSSO algorithm is a blend of SSO and PSO algorithms. The parameter settings utilized in the numerical experiments are based on the selection of the following parameters.

For PSO method: $P$ = 300 (maximum population size), $N$ = 4 (number of iterations), $\omega$ = 0.9 (inertia weight), $C_{1}$ = 2.05 (personal learning coefficient) and $C_{2}$ = 2.05 (global learning coefficient).

For SSO method: $P$ = 300 (maximum population size), $N$ = 4 (number of iterations), $L_{b}$ = 0.0 (lower bound of dimension), $U_{b}$ = 0.98 (upper bound of dimension). The inertia weight is used to balance the global search and the local search. The value of the inertial coefficient $\omega$ is typically between 0.8 and 1.2, which can either dampen the particle’s inertia or accelerate the particle in its original direction. Generally, lower values of the inertial coefficient speed up the convergence of the swarm to optima, and higher values of the inertial coefficient promote exploration of the entire search space. $C_{1}$ and $C_{2}$ are called learning factors or cognitive coefficient and social coefficient respectively. The cognitive coefficient $C_{1}$ is usually close to 2 and affects the size of the step the particle takes toward its individual best candidate solution (pbest) found around the search space. The social coefficient $C_{2}$ is typically close to 2 and represents the size of the step the particle takes toward the global best candidate solution (gbest) found around the search space. $C_{1}$ and $C_{2}$ control the levels of exploration and exploitation. Exploitation is the ability of particles to target the best solutions found so far. On the other hand, exploration is the ability of particles to evaluate the entire research space. In this study, the two learning factors $C_{1}$ and $C_{2}$ have the same value of 2.05, so that the social and cognitive searches have the same weight. The choice of population size is related to the problems to be solved, but it is not very susceptible to the concern problems. In this study, a larger population of 300 is used for both PSO and SSO to meet the specific requirements. The population is created between lower bound Lb and upper bound Ub of the search space that is selected as 0.0 and 0.98 respectively.

The input DC-DC buck-boost converter inductor of 80 μH and the output capacitor of 220 μF are chosen with the switching frequency fp = 50 kHz. The proposed PSOSSO parameters are combined PSO, and SSO parameters.

*Case 1* Hardware implementation, the converter voltage, current and battery voltage, current, power, and efficiency were presented in light weather varying conditions at 25 °C and depicted in Figs. 8, 9, and Fig. 10. In this case 1, the simulations are done by using the above PSOSSO parameters and the solarland PV module (SLP190S-24).

**Figure 7:**
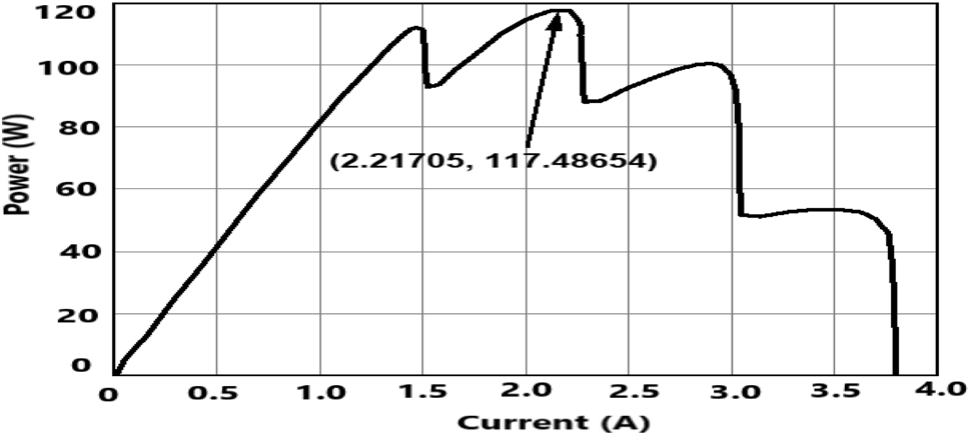
The P–I curve under partial shading condition (PSC).

*Case 2* Performance of the proposed PSOSSO algorithm is compared with conventional techniques PO, FA, DE, PSO, SSA, and ISSA algorithms. The results are presented and discussed in Figs. 11, 12, and 13, and Tables 1, and 2 based tracking time, settling time, power convergence. To accurately compare and confirm the validity of the proposed PSOSSO method with the other conventional techniques mentioned above, the simulations were carried out by using^38^ system parameters, PV module (MAX60), DC-DC buck-boost converter, and operating conditions such as: sampling time, switching frequency, irradiance change range, temperature and duty cycle. Through this comparison, we conclude that the proposed PSOSSO succeeded in extracting MPP of 0.1667Kw at 0.19 s of tracking time. The obtained curves of PSOSSO and other conventional methods are depicted in Figs. 12 and 13. The results show that the proposed PSOSSO method has tracked higher power, fast-tracking time, good settling time, without oscillation, more accurate with higher efficiency compared to PO, FA, PSO, DE, SSA, and ISSA methods. The P-I curve under partial shading condition (PSC) is depicted in Fig. 7 with a global maximum power of 0.11748 KW.

**Table 2:** Comparative analysis in terms of various criteria^37^.

Criteria	PSOSSO	ISSA	SSA	FA	DE	P&O
Periodic tuning	Not required	Not required	Not required	Not required	Not required	Not required
Tracking accuracy	Highly accurate	High	High	High	Accurate	Low
Steady-state oscillation	Zero	Zero	Zero	Zero	Zero	High
Tracking speed	Fast	Medium	Medium	Medium	Medium	Very fast
Algorithm complexity	More	Medium	Medium	Medium	Medium	Simple
Efficiency	High	Average	Average	High	Average	Very poor
Computational time	2.33 s	2.39 s	2.58 s	2.46 s	3.19 s	3.38 s

Based on the transient stability (overshoot) of the selected methods, the proposed PSOSSO is ranked at the forefront followed by ISSA, then SSA, then PSO, then DE, then P&O, and lastly by FA.

As for the DE algorithm, it has a crossover operator which prevents the candidate solution from stucking at a local optimal position as compared to the P&O algorithm and its mutation process is more effective in diversifying the candidate solutions as compared to the local version (search space) in PSO algorithm.

As for the FA algorithm, moreover, its absorption process, it has three main components such as the randomization parameter, the attractiveness, and the absorption coefficient. The attractiveness depends on the brightness (light intensity) of the firefly agent which can cause a random walk when the brighter firefly is not found so far, thus reducing its optimization performance. As a result, the mutation and crossover features of DE can help to circumvent this weakness. However, the SSA has a mediocre convergence rate and limited exploratory capabilities, its ability to improve the quality of the population proportional to the number of the iterations and the PSO strategy strengthen the proposed PSOSSO algorithm to set a balance between the exploration and the exploitation of the SSO and increase the convergence rate. Although the two combined parameters of SSO and PSO make the proposed PSOSSO algorithm more complex, their features improve the optimization performance of the proposed PSOSSO with higher tracking efficiency of 99.52% and a lower computational time of 2.33 s as compared to the other conventional techniques.

In terms of tracking efficiency after the proposed PSOSSO technique in decreasingly ranked manners, ISSA has 99.13%, SSA with 99.04%, FA with 99.01%, DE with 98.89%, and lastly P&O with 92.44%.

For the computational time after the proposed PSOSSO technique in decreasingly ranked manners, ISSA has 2.39 s, FA with 2.46 s, SSA with 2.58 s, DE with 3.19 s, and lastly P&O with 3.38 s.

The DE, FA, SSA, and ISSA algorithms are not cumbersome in complexity as compared to the PSOSSO algorithm.

The P&O is the simplest algorithm, and it has the lowest optimization performance and is less stable as compared to other techniques.

*Case 3* Energy studies in various seasons comparison were carried out among the proposed PSOSSO algorithm and the existing P & O, ABC, and CS algorithms and presented in Fig. 14 and Table 4.

*Case 4* The partial shading condition (PSC) was applied to the proposed PSOSSO algorithm, and the results were presented in Fig. 15.

*Case 5* Complex Partial shading (CPS) scenarios are elaborated, and the simulation results are shown in Fig. 16.

This scenario is composed of a 4SP3 pattern of 4 series raw and 3 parallel columns. The scenario is called complex partial shading (CPS) because each module has several irradiances of the same values or different values and is connected to form a pattern. In this study, patterns are defined as follows.

Pattern#1: G1 = G2 = G3 = G4 = [1000, 1000, 1000, 1000] W/m^2^ applied in time interval of [0, 0.3, 0.6, 0.8] s.

Pattern#2: G1# G2# G3# G4 = [1000, 800, 600, 300] W/m^2^ applied in time interval of [0, 0.3, 0.6, 0.8] s.

Pattern#3: G1# G2# G3# G4 = [900, 700, 500, 200] W/m^2^ applied in time interval of [0, 0.3, 0.6, 0.8] s.

The simulation results are depicted in Fig. 16a and b with battery and resistor as load respectively.

### Case 1: normal operation with varying weather change conditions

**Figure 8:**
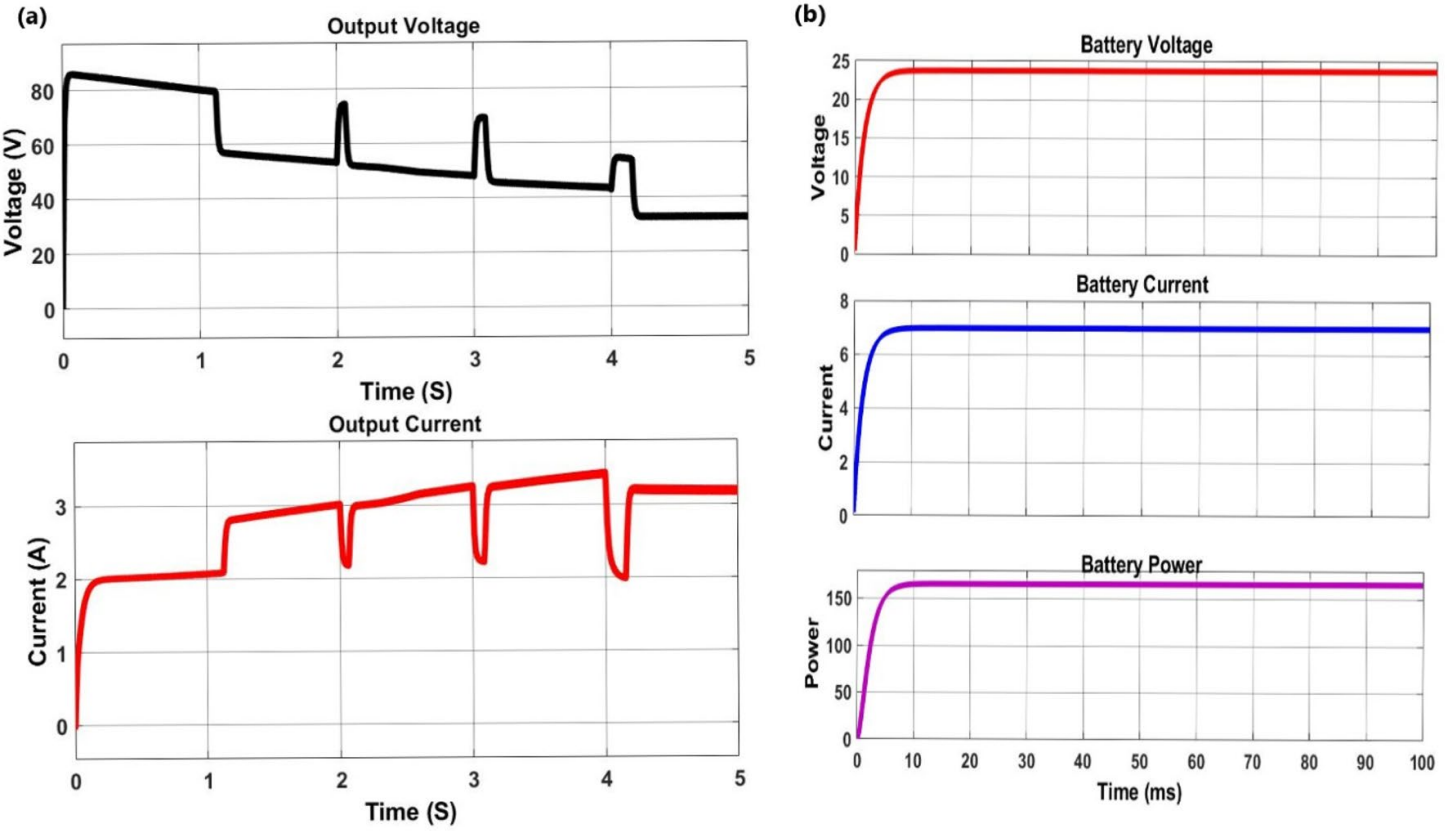
The converter output voltage, and output current are presented because as the converter is dealing with a large load (battery) step from the discontinuous current mode (DCM) to the continuous current mode (CCM), therefore; the transient condition is important (**a**). Battery (Load) current, voltage, and power (**b**).

Figure 8a, shows that the isolated buck converter output voltage increases and the output load current decreases. Figure 8b depicted: Battery Power, current, and voltage. The efficiency is constant in all stages and hence the buck-boost converter is stable in all conditions.

**Figure 9:**
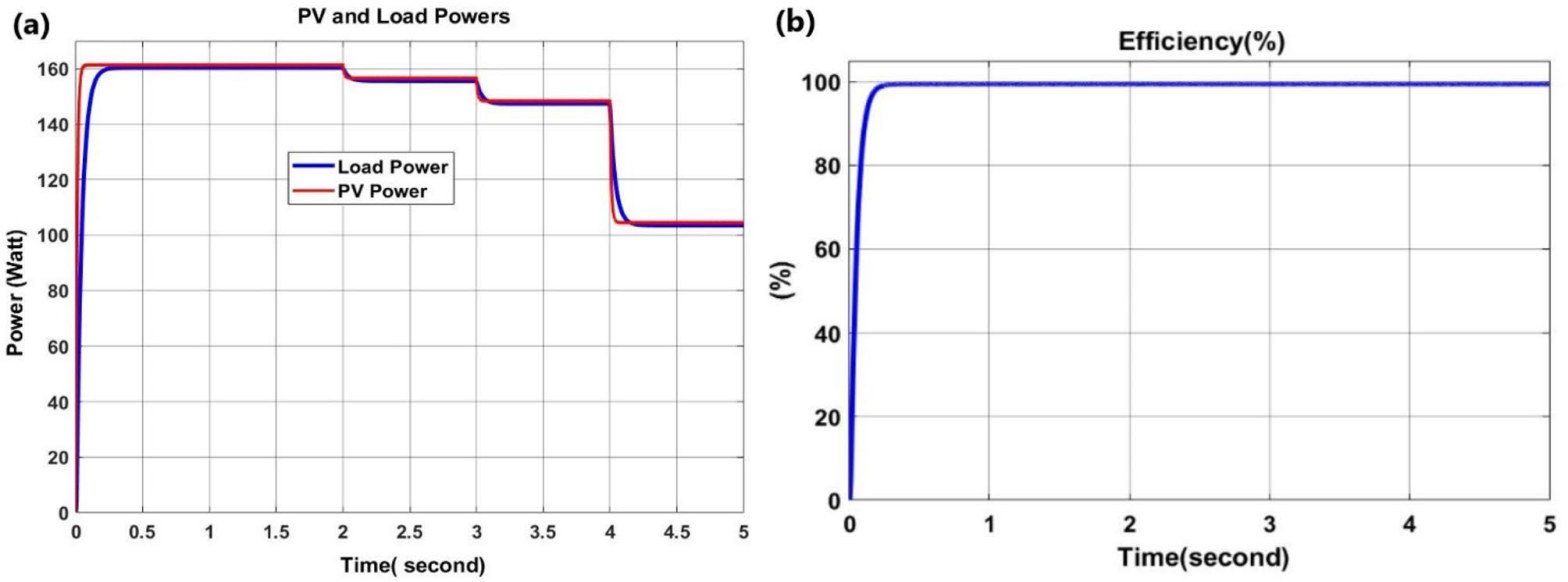
PV and battery (load) powers (**a**) and efficient of 99.99% at standard test conditions (**b**).

From the above Fig. 9, the input PV power is in the same tracking of the load power by irradiation variation of [0,1000]; [1000,900]; [900, 800] and [800, 600] W/m2 in time interval of [0,2]; [2,3]; [3,4] and [4,5] second respectively at 25 °C. The power value is constant since the voltage is inversely proportional to the current and hence the power constant is shown. The input power and output power are in the same tracking as result.

**Figure 10:**
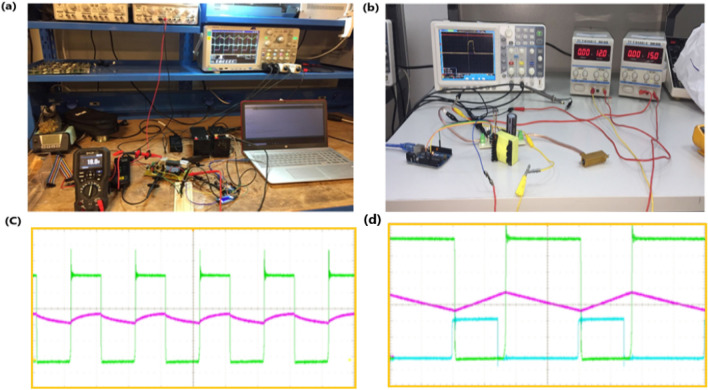
Hardware setup of the proposed system (**a**), the buck-boost converter and inductor design (**b**) the buck-boost converter Mosfet drain-source voltage (green) and output current (purple) (**c**) and the Mosfet drain-source voltage (green), inductor current (purple) and microcontroller PWM (blue) (**d**).

Figure 10c and d are the implementations of results of load (battery) current and voltage, input current and input voltage, buck-boost converter inductor current, and duty cycle which confirm the accuracy of the implementations in real-time where the implemented components are shown in Fig. 10 above.

### Case 2: comparative analysis in terms of various parameters

The proposed hybrid PSOSSO is analyzed by comparing it with the existing methods Improved Salp Swarm Algorithm (ISSA), Firefly Algorithm (FA), Salp Swarm Algorithm (SSA), Differential Evolution (DE) and Perturbation, and Observation (P&O) in terms of periodic tuning, tracking accuracy, steady-state oscillation, tracking speed, algorithm complexity, and efficiency. It is shown in below Table 2.

**Figure 11:**
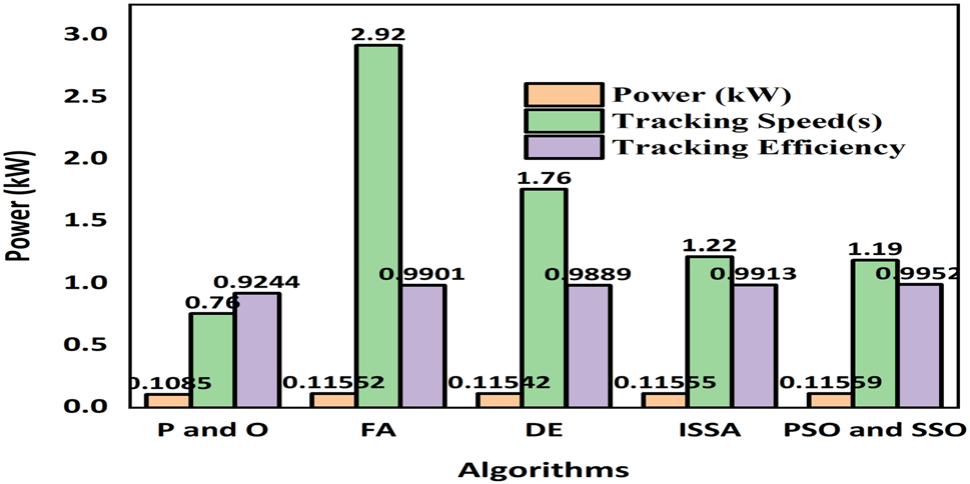
Comparative analysis of the proposed and existing methods^37^ in terms of various performance metrics.

From the above Table 2, the analysis of the performance of the existing methods and the proposed method in terms of the mentioned criteria are shown. It is found that the proposed hybrid PSOSSO does not require periodic tuning, highly accurate tracking, zero steady-state oscillation, fast-tracking speed, high algorithm complexity, low computation time, and high efficiency. On the other hand, the existing methods are effective only in some cases, whereas the proposed methodology shows effective performance than the existing methods in terms of all the mentioned criteria. The proposed hybrid PSOSSO is also analyzed in terms of power, tracking speed, and tracking efficiency depicted in Fig. 11 below. The results are shown in below Table 3.

**Table 3:** Comparative analysis in terms of various parameters^37^.

Algorithm	Power (KW)	Tracking speed (s)	Tracking efficiency (%)
P&O	0.1085	0.76	92.44
FA	0.11552	2.92	99.01
DE	0.11542	1.76	98.89
SSA	0.11558	1.53	99.04
ISSA	0.11559	1.22	99.13
PSOSSO	0.11667	0.19	99.52

From the above Table 3, it is clear that in partial shading conditions, the proposed PSOSSO method shows power at a rate of 0.11667 KW, tracking speed at a rate of 1.19 s, and tracking efficiency at a rate of 99.52%. On the other hand, all other existing methods exhibited better results in terms of power, tracking speed, and tracking efficiency. But, when the existing methods are compared with the proposed system, the results reveal that the proposed system outperforms other existing methods that prove the efficiency of the proposed methodology. It is graphically shown in the below Fig. 11.

**Figure 12:**
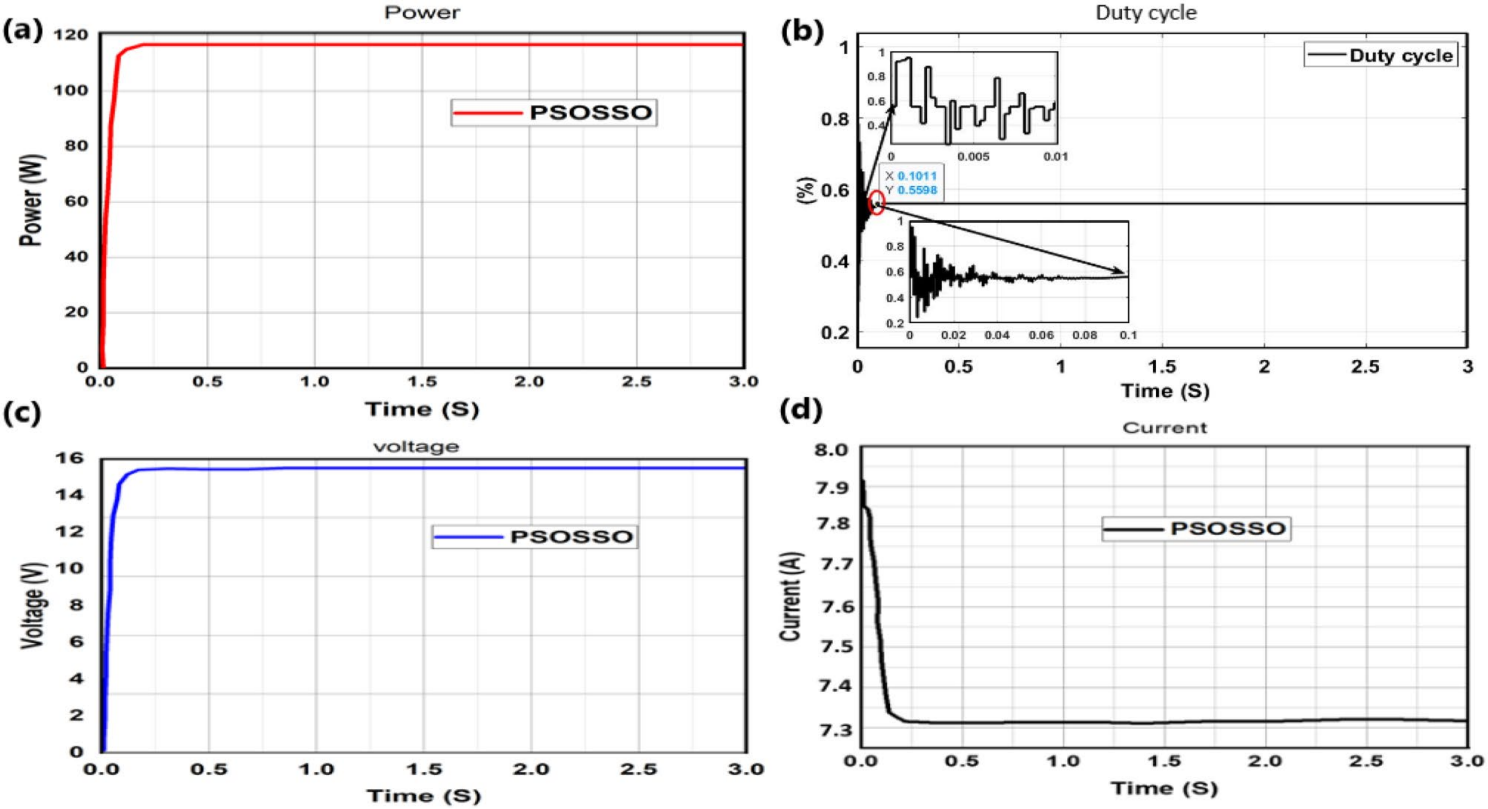
Tracking mechanism of PSOSSO under partial shading condition with linear load (resistor) connected.

**Figure 13:**
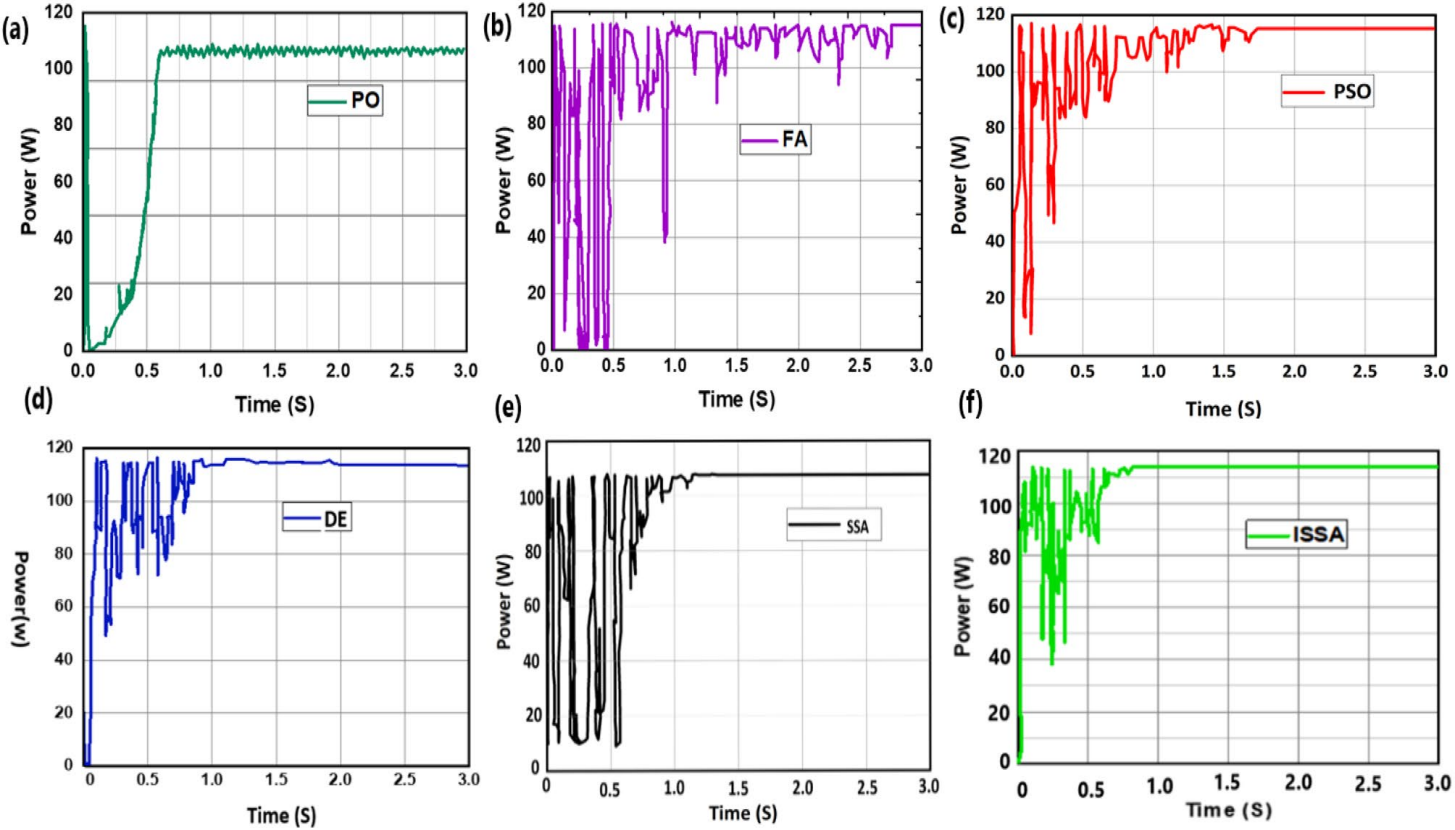
Tracking mechanism of P&O, FA, PSO, DE, SSA, and ISSA under partial shading condition with linear load (resistor) connected.

The proposed technique PSOSSO has successfully succeeded to track the higher power of 0.11667 KW with a lesser tracking time of 0.19S and the magnitude of oscillation rate is quite reduced Fig. 12a as compared to the other techniques depicted in Fig. 13. The duty cycle strikes a value of 0.5598 at 0.1S and remains steadily constant until the end time of 3S Fig. 12b, inside this Figure the magnified duty cycle at [0 0.01] second and [1 0.1] second are presented at left upper and lower corner respectively. The voltage seated at [15.93 V 0.231S] Fig. 12c, and current at [7,321A, 0.275S] Fig. 12d.

The simulation results show in descending order the power extracted by each algorithm follow by their tracking time as: ISSA (115, 94 W, 0.81 S), SSA (0.11589 KW, 1.16 S), PSO (0.11522 KW, 1,73 S), FA (0.11520 KW, 2,76 S), DE (0.11513 KW, 1.62 S), P&O (0.10857 KW, 0.6 S). The highest efficiency of 99.52% is achieved by PSOSSO Fig. 12a, followed 99.13 by ISSA Fig. 13f, by 99.12 SSA Fig. 13e, by 99.04 PSO Fig. 13c, by 99.01 FA Fig. 13b, by 98.89 DE Fig. 13d, and the lowest efficiency of 92.44 by P&O Fig. 13a.

### Case 3: comparative analysis in terms of various parameters

**Figure 14:**
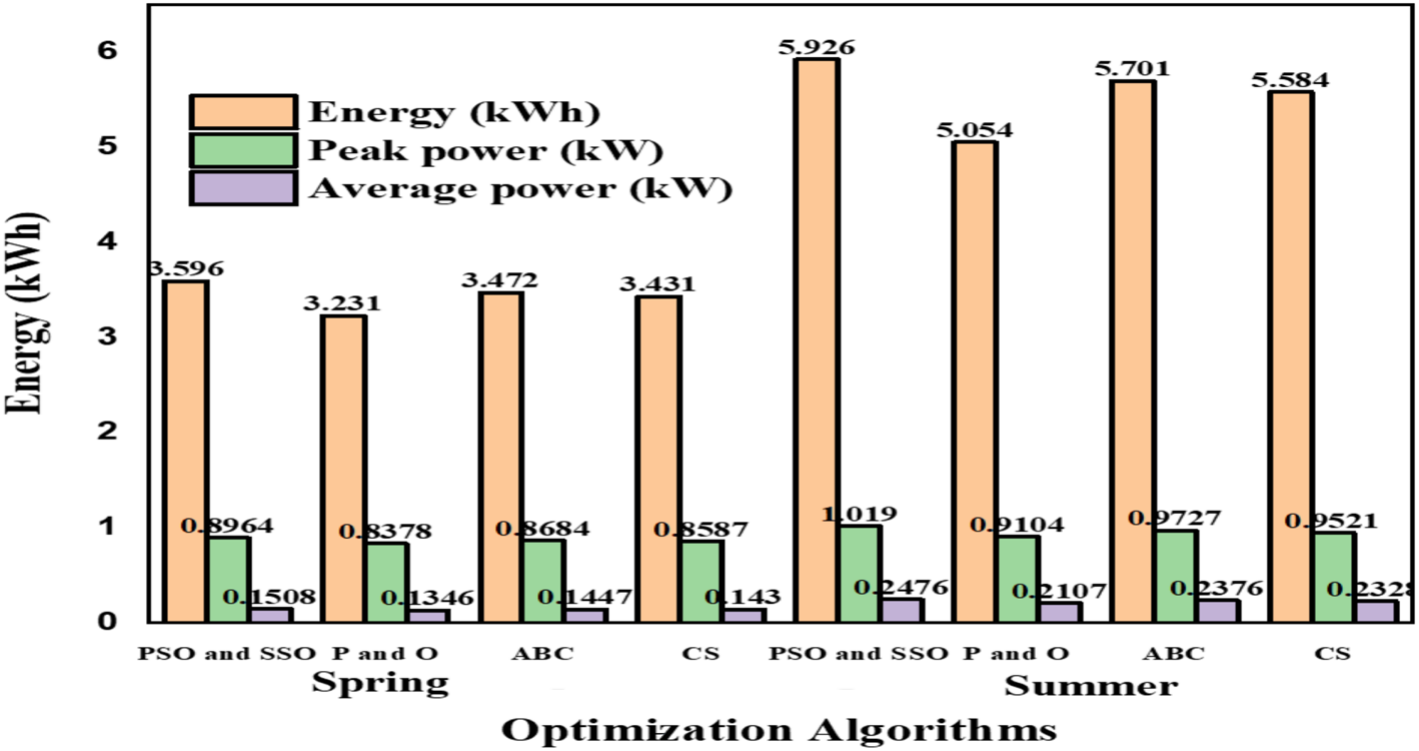
Comparative analysis for battery charging in various seasons^38^.

From the above Table 4, It is found that the existing P&O, ABC, and CS algorithm shows low performance than the proposed method, and hence It is clear that the proposed method is better than the existing method in spring and summer concerning energy, peak power and average power depicted in Fig. 14 above.

**Table 4:** Comparative analysis in various seasons^38^.

Season	Measure	PSOSSO	P & O	ABC	CS
Spring	Energy (KWh)	3.596	3.231	3.472	3.431
	Peak power (KW)	0.8964	0.8378	0.8684	0.8587
	Average power (KW)	0.150.8	0.1346	0.1447	0.143
Summer	Energy (KWh)	5.926	5.054	5.701	5.584
	Peak power (KW)	1.019	0.9104	0.9727	0.9521
	Average power (KW)	0.2476	0.2107	0.2376	0.2328

### Case 4: partial shading conditions of 4S2P pattern

**Figure 15:**
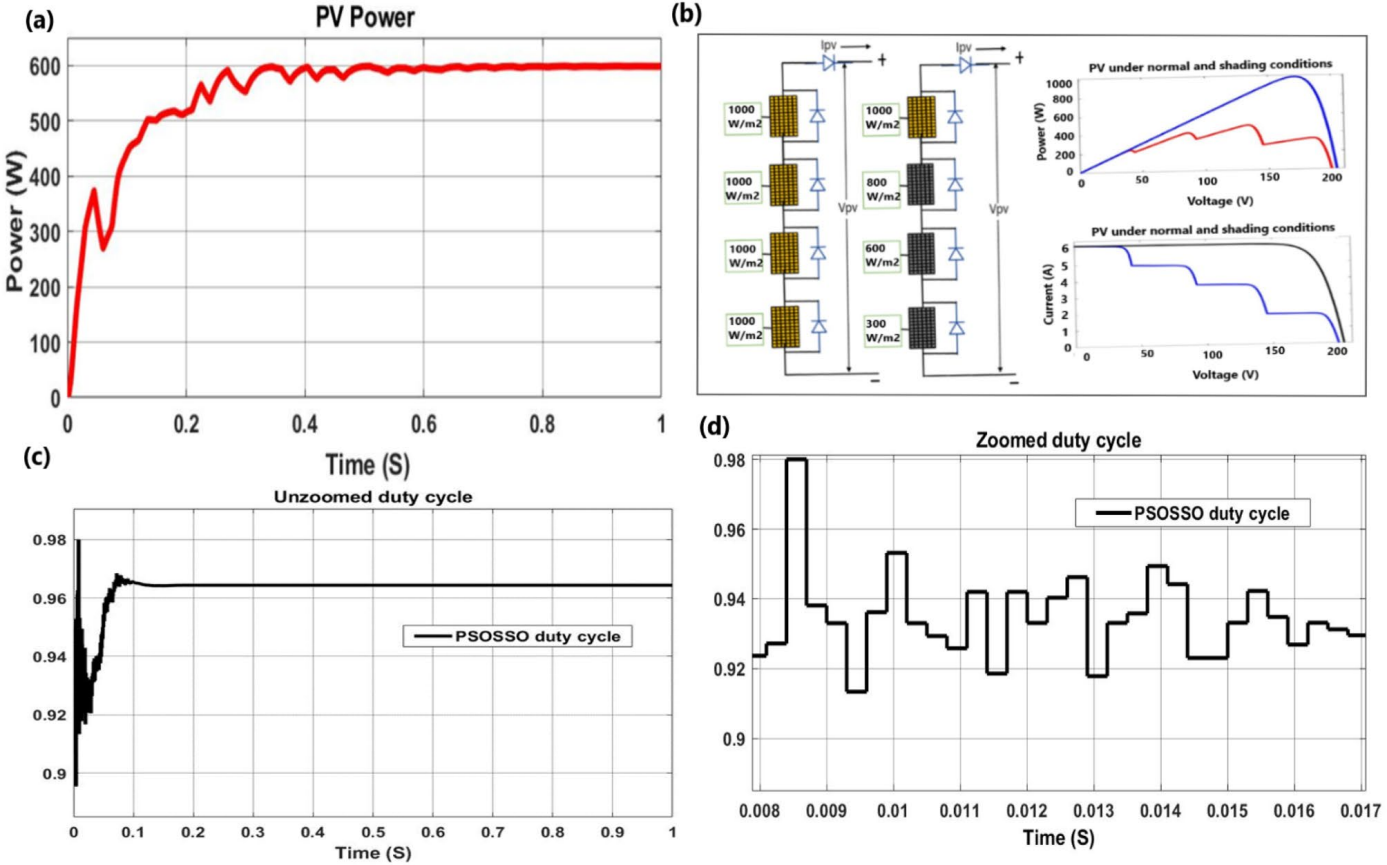
Tracking mechanism of PSOSSO under partial shading condition with nonlinear load (battery) connected.

The proposed method extracts maximum power of 600 W at 0.8 S. It has a good duty cycle of 0.91 under PSCs, but its performance is slightly affected by the nonlinear characteristics of the battery which presents some oscillations from 0 to 0.8 s Fig. 15a. The 4S2P pattern irradiation on each module (G1 = [1000, 1000, 1000, 1000] W/m2 and G2 = [1000, 800, 600, 300] W/m^2^) of PV module and P–V, I-V curves under normal, and partial shading conditions at 25 °C Fig. 15b. Non magnified duty cycle with a steady value of 0.965 from 0.1 to 1 s Fig. 15c. The magnified duty cycle was extracted from Fig. 15c at an interval of [0.008 0.017] second with an average value of 0.91 Fig. 15d.

### Case 5: complex partial shading conditions (CPSCs) or clusters

**Figure 16:**
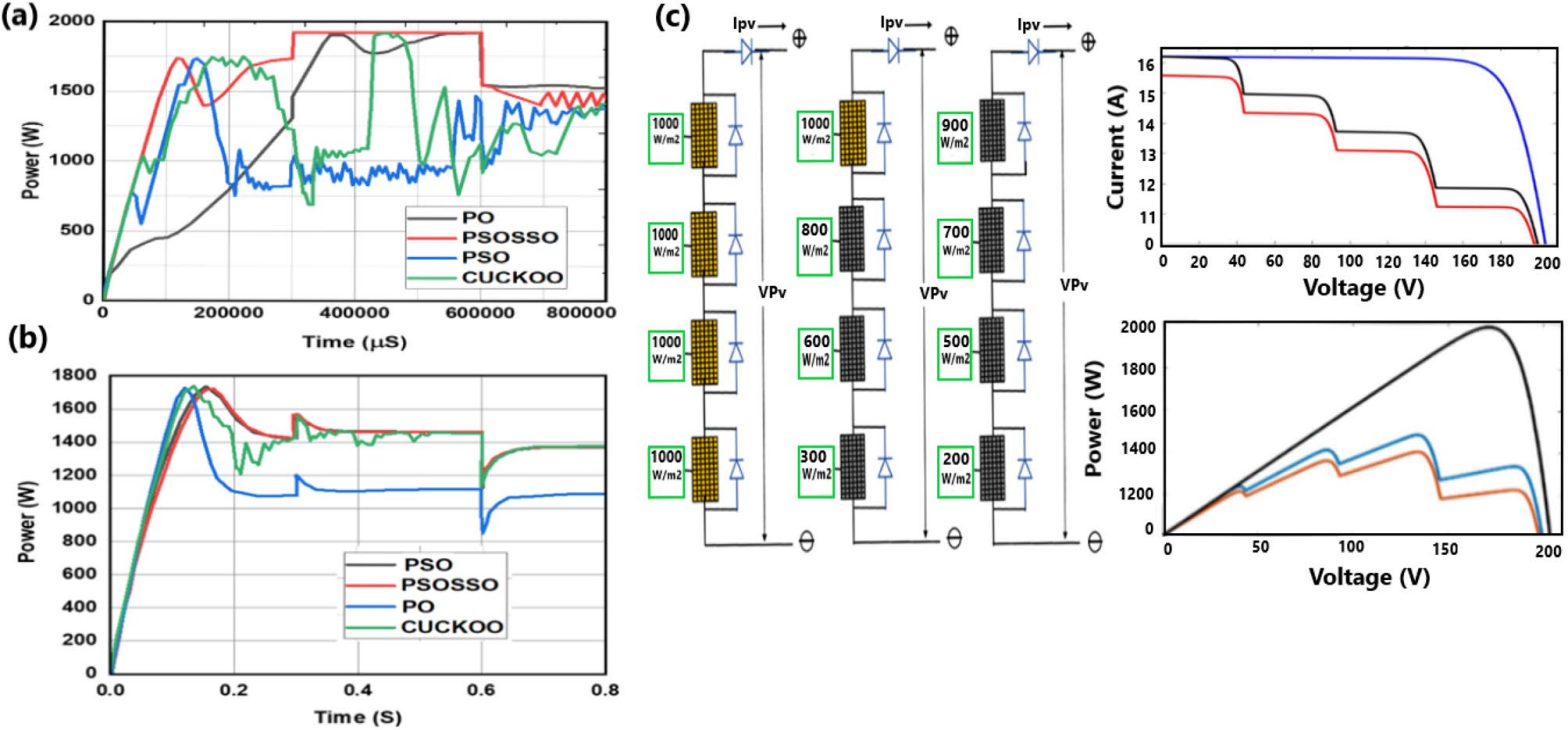
Tracking mechanism of PSOSSO under complex partial shading conditions (CPSCs) condition with linear load (resistor) (**b**) and nonlinear load (battery) (**a**) connected. The partial shading Conditions (4SP3) PV module connection and the P–V, I–V curves (**c**).

The proposed PSOSSO algorithm highly and accurately performs with better power tracking when a linear load (resistor) or nonlinear load (battery) is connected. The nonlinear characteristics of the battery affect the system performance, however, PSOSSO has higher power and better efficiency as compared to other techniques. In Fig. 16a, PSOSSO presents a good transient shape (minimum overshoot) at settling time from 0 to 100,000 µS, and abruptly it drops in power from 100,000 to 300,000 µS where the duty cycle is stablishing from its changing conditions. At 300,000 µS to 600,000 μS, a rapid increase in power is observed. From 600,000 to 800,000 µS, it presents some oscillations at the battery’s full charging state. The observation shows that the other conventional present low power with a large oscillation range. In Fig. 16b, at 0 to 0.1 S (lower-left corner zoomed Fig. 12b), where the duty cycle is changing its state, the power decrease is observed from the PSOSSO method. At 0.1 S to 0.8 S where the duty cycle is constant, PSOSSO successfully tracks a global maximum power (GMP) with less fluctuation as compared to PO, PSO, and CUCKOO techniques. The PSOSSO and PSO have higher powers with oscillation free whereas P&O and CUCKOO have lower power with oscillation and oscillation free respectively.

Overall simulation results in partial shading conditions (PSCS) and complex partial shading conditions (CPSCs) have proved that the proposed PSOSSO algorithm demonstrates higher performance and good accuracy as compared to the existing techniques due to the characteristics of the PSO strategy enhancing the SSO ability for exploration and exploitation with the quality improvement of the SSO in the searching space for the candidate solutions. These specific features of the PSOSSO algorithm improve its efficiency without affecting the computational effort as compared to other techniques.

## Conclusion

A novel PSOSSO based MPPT mechanism is proposed in this paper for extracting the global maximum power (GMP) from the PV system. The proposed PSOSSO technique has been compared with the renowned existing MPPT techniques. Five (5) different cases are used to perform the comparison of the new technique with the old ones. The simulation results show that the proposed method outperforms the conventional existing techniques in terms of power, tracking time, and tracking efficiency and robustness. Depending on the operation conditions such as partial shading condition (PCS) or complex partial shading condition (CPSC) with linear (resistor) or nonlinear (battery) load connected, the proposed PSOSSO technique is capable to track the global maximum power (GMP) within 0.1–0.19 S as compared to PSO, FA, DE, SSA, ISSA, P&O, and CUCKOO techniques. The ability of fast GMP tracking, and short settling time (with less overshoot) enable PSOSSO to maximize power in the transient state. Moreover, the PSOSSO has oscillation-free features with reduced overshoot and ripples in complex partial shading conditions as compared to other methods.

The future work can be the hardware implementation of charging electric vehicles by using the proposed approach (Supplementary Tables).

## Supplementary Information

Supplementary Tables.
