# Supplementary material for: MPPT mechanism based on novel hybrid particle swarm optimization and salp swarm optimization algorithm for battery charging through simulink
Source: Sci Rep. 2022 Feb 17;12:2664. doi: 10.1038/s41598-022-06609-6 (PMC8854737; doi:10.1038/s41598-022-06609-6)
Supplement: Supplementary file 1 — Supplementary Tables. [file 41598_2022_6609_MOESM1_ESM.pdf]

Appendix

Table A1. Electrical parameters of SLP190S-24 PV solar module

PV Module Electrical Characteristics	Symbol	SLP190S-24
Product Code	-	19002240ID
Maximum power	Pmax	190W
Voltage at Pmax	Vmp	36.8 V
Current at Pmax	Imp	5.16 A
Open-circuit Voltage	Voc	45 V
Short-circuit current	Isc	5.56 A
Temperature coefficient of Voc	Tvoc	-(80 ∓ 10) mV/ºC
Temperature coefficient of Isc	TIsc	(0.065±0.0015) %/ºC
Number of cells connected in series	Ns	36

Table A2. Buck-boost converter parameters

Designation	Symbol	Values
Inductor (EE42)	L	80 μH
Input capacitor	Cin	220μF
Output capacitor	Co	220 μF
Switching frequency	fp	50KHz
Mosfet ((IRF3710pbF N channel)	Q1	-
Diode (MBR2015OCT)	D1	-
Resistor load	R	50 Ω

Table A3. Technical specification of the battery

Designation	Symbol	Values
Battery type (Lead acid)	Maintenance free	AGM Technology
Rated voltage	V	24V
Number of elements	PCs	6
Capacity	Q	7 Ah
Internal resistance	Rin	3.4Ω
Weight	W	62Kg
Short circuit current	Isc	3400 A
